# EBV-related lymphoma after long-term daratumumab treatment: a case report

**DOI:** 10.1038/s41408-020-00379-y

**Published:** 2020-11-04

**Authors:** Ilse P. G. Verpoorte-Botden, Monique C. Minnema, Reinier A. P. Raymakers

**Affiliations:** 1 Department of Hematology, University Medical Center Utrecht, Utrecht, The Netherlands; 2 Department of Internal Medicine, Ziekenhuis Rivierenland Tiel, Tiel, The Netherlands

**Keywords:** Adverse effects, Myeloma, B-cell lymphoma, Cancer immunotherapy

Dear Editor,

The CD38 antibody daratumumab is effective multiple myeloma (MM) treatment^1–3^ and as such registered both as monotherapy and as in combination therapy by the U.S. Food & Drug Administration and the European Medicines Agency for both patients with newly diagnosed MM who are not eligible for autologous stem cell transplantation (SCT) as well as for patients with relapsed/refractory MM.

In this report, we describe a patient who was included in phase I/II clinical study with daratumumab (in combination with lenalidomide and dexamethasone; see for details ref. ^4^) and who developed an Epstein–Barr virus (EBV)-related lymphoma; possibly associated with the long-term use of daratumumab.

Patient A was diagnosed with MM (IgG lambda, 13 g/l, ISS stage II, CRAB criterion: osteolytic lesions; no cytogenetics available) at the age of 69. He reached a complete response (CR) after 3 cycles of thalidomide-doxorubicin-dexamethasone (TAD), cyclophosphamide–doxorubicin–dexamethasone (CAD), and was consolidated by high-dose melphalan (HDM) and autologous SCT. Already 5 months after HDM he showed progression and was treated with bortezomib–dexamethasone (and temporary thalidomide). Eight months after the start of this line of therapy, he progressed again and was included in the GEN503 study. Treatment commenced with daratumumab (8 mg/kg, intravenous weekly for the first two cycles, infusions every other week for cycles 3–6, then once per cycle), in combination with lenalidomide (25 mg; days 21–28) and dexamethasone (40 mg weekly, cycles of 28 days), which resulted in a stringent CR after 8 cycles. After 40 months and still on treatment, a positron emission tomography (PET) scan (performed because of backache but without other symptoms) demonstrated fluorodeoxyglucose (FDG) avid lymphadenopathy cervical, axillary, mediastinal, retroperitoneal, inguinal, and an FDG avid enlarged spleen. An excision biopsy of a cervical lymph node showed a polymorph lymphoproliferative disorder (LPD) in the context of immune suppression (iatrogenic immune deficiency associated LPD), and together with slightly elevated EBV plasma-PCR values (208 IU/ml; which were negative before), an EBV-related lymphoma was diagnosed. Daratumumab, lenalidomide, and dexamethasone were discontinued. The lymphoma was treated with 4 cycles of rituximab 375 mg/m^2^ at weekly intervals, which resulted in metabolic CR and negative EBV PCR. Currently, 3 years later, both the MM and the lymphoma are still in CR without any further treatment.

As the first clinical trials with daratumumab started in 2008, few data are available on long-term complications of daratumumab treated patients^4,5^. We describe a potentially serious hematologic complication during continued daratumumab treatment in combination with lenalidomide and dexamethasone, i.e., the development of an EBV-related lymphoma, considered a rare complication after therapy for MM.

EBV-related lymphoma is associated with an acute EBV-infection (in both Hodgkin and Burkitt lymphoma) but can also develop as post-transplantation lymphoproliferative disorder (PTLD) as a result of extrinsic immunosuppression after the organ or allogenic SCT^6^. Furthermore, it has been described in patients with auto-immune disease^6^ and after autologous SCT^7^ and is then referred to as (iatrogenic) LPD. PTLD and LPD may be initiated when an impaired cytotoxic T-cell response fails to control the proliferation of EBV-infected cells (other than naive B-cells in the Waldeyer’s ring)^6^. They represent a spectrum of EBV-related diseases, from an early polyclonal mononucleosis-like illness (non-destructive) to polymorphic, monomorphic, and classic Hodgkin lymphoma LPD^8^. Treatment of EBV-related lymphoma combines the reduction of immunosuppression, administration of rituximab (B-cell-specific antibody against CD20), and sometimes chemotherapy^9^.

EBV-related lymphoma has been described twice after treatment with daratumumab. In the POLLUX trial, one patient discontinued daratumumab-lenalidomide-dexamethasone because of an EBV-associated lymphoma^10^, and in the GEN503 study, one patient died due to respiratory insufficiency resulting from polymorphic PTLD^11^. No clinical cases of EBV-related lymphoma after lenalidomide are described. McCarthey et al. found an increased incidence of Hodgkin lymphoma after lenalidomide maintenance although no infection or reactivation of latent EBV was demonstrated^12^. However, in vitro data show that lenalidomide with or without glucocorticoids may reactivate EBV-positive resting memory B-cells^13,14^.

The pathogenesis in this patient could be the immune suppression by daratumumab, perhaps in combination with lenalidomide and dexamethasone comparable to immunosuppression after transplantation. Indeed, at the time of diagnosis of the EBV-related lymphoma, a lymphopenia was present (0.53 × 10^9^/l), and stopping daratumumab together with 4 doses of rituximab cured the lymphoma, comparable to PTLD. In line with this immune-suppressive mechanism is the described symptomatic cytomegalovirus (CMV) reactivation in a patient during daratumumab monotherapy^15^ and severe CMV-related gastro-intestinal disease in three patients receiving daratumumab^16^.

In conclusion, we describe a secondary hematological malignancy in a patient after prolonged administration (i.e., more than 3 years) with daratumumab, in combination with lenalidomide and dexamethasone. Daratumumab has demonstrated to be an effective therapy in MM, however, possibly long-term continuation of treatment up to progression may include a risk for late complications. Since the combination daratumumab, lenalidomide and dexamethasone are prescribed more frequently and also used in frontline treatment of MM^17^, apparently leading to longer remission free periods, awareness of possible late complications is important.
